# Comprehensive approach to weaning in difficult-to-wean infantile and juvenile-onset glycogen-storage disease type II patients: a case series

**DOI:** 10.1186/s13052-019-0692-0

**Published:** 2019-08-22

**Authors:** Lingling Xu, Hongjun Ba, Yuxin Pei, Xueqiong Huang, Yujian Liang, Lidan Zhang, Huimin Huang, Cheng Zhang, Wen Tang

**Affiliations:** 1 Department of PICU, The First Affiliated Hospital, Sun Yat-Sen University, 58 Zhongshan Second Road, Guangzhou, Guangdong 510080 People’s Republic of China; 2 Department of Cardiovascular pediatrics, The First Affiliated Hospital, Sun Yat-Sen University, 58 Zhongshan Second Road, Guangzhou, Guangdong 510080 People’s Republic of China; 3 Department of Neurology, The First Affiliated Hospital, Sun Yat-Sen University, 58 Zhongshan Second Road, Guangzhou, Guangdong 510080 People’s Republic of China

**Keywords:** Enzyme replacement therapy, Glycogen storage disease type II, Respiratory failure, Sequential invasive-noninvasive ventilation, Weaning

## Abstract

**Background:**

Glycogen storage disease type II (GSD II) is caused by acid alpha-glucosidase (GAA) deficiency. Both infantile-onset and juvenile-onset GSD II lead to proximal muscle weakness and respiratory insufficiency and require mechanical ventilation. However, GSD II is also independently associated with delayed weaning from mechanical ventilation. This study aimed to describe a comprehensive approach including sequential invasive-noninvasive mechanical ventilation weaning and enzyme replacement therapy (ERT) in patients with weaning difficulties.

**Case presentation:**

We studied six difficult-to-wean GSD II (three juvenile-onset, three infantile-onset) patients at the First Affiliated Hospital, Sun Yat-sen University from October 2015 to December 2017. Difficulty in weaning was defined as follows: the need for more than three spontaneous breathing trials or more than 1 week to achieve successful weaning. All patients received comprehensive treatment including sequential invasive-noninvasive mechanical ventilation weaning, ERT and general treatment. Recombinant human acid alpha-glucosidase enzyme therapy (20 mg/kg every 14 days) was used after diagnosis, and Patients 1–6 received ERT for 15.5, 4.5, 2, 2.5, 17, and 2 months, respectively. The therapeutic effect of the comprehensive treatment was observed.

The patients’ respiratory function and limb muscle strength improved after each ERT session. Patients who successfully completed a spontaneous breathing trial could proceed to extubation, and then start non-invasive ventilation. The patients’ age range at initial mechanical ventilation was 3–47 (median 26.5) months, duration of invasive ventilation was 1–36 (median 2.75) months, and duration of noninvasive ventilation was 0–0.6 (median 0.05) month. The patients’ nutritional status improved after enhanced nutritional support. Patients 2, 3, and 5 were successfully weaned off the ventilator. Patient 1 underwent tracheal intubation after six weaning failures, and Patients 4 and 6 died after therapy was abandoned by their parents.

**Discussion and conclusions:**

Male sex, GSD II type, and the presence of malnutrition and neurological impairment may predict poor respiratory outcomes. The above-described comprehensive sequential invasive-noninvasive mechanical ventilation weaning strategy may increase the success rate of weaning from mechanical ventilation.

## Background

Glycogen storage disease type II (GSD II), also known as Pompe disease, is an autosomal recessive inherited metabolic disorder caused by a deficiency of acid alpha-glucosidase (GAA); it manifests as muscle weakness, hypertrophic cardiomyopathy, and respiratory failure [1]. GAA deficiency leads to systemic glycogen accumulation in the lysosomes of skeletal muscle, motor neurons and smooth muscle, which causes progressive proximal muscle and respiratory muscle weakness. GSD II is categorized into early-onset (infantile-onset) and late-onset (juvenile-onset and adult-onset) GSD II by the age at onset. Infantile-onset GSD II is the most severe form of the disease, and is characterized by hypertrophic cardiomyopathy, muscle weakness and early death, generally caused by cardio-respiratory insufficiency as natural history. Both infantile-onset and juvenile-onset GSD II lead to proximal muscle weakness and respiratory insufficiency and require mechanical ventilation. Limb weakness, respiratory muscle weakness and diaphragm weakness are independently associated with delayed weaning from mechanical ventilation [1–3]. Prolonged mechanical ventilation is known to result in increased pulmonary complications and costs [4], as well as increased lengths of hospital stay and clinical scores.

Enzyme replacement therapy (ERT) cannot improve muscle strength and respiratory function, and result in weaning from the ventilator in all patients. Recombinant human acid alpha-glucosidase enzyme therapy can improve patients’ muscle strength and respiratory function [5, 6]. Sequential invasive-noninvasive ventilation is useful in treating patients with invasive mechanical ventilation weaning failure [7]. Additionally, symptomatic and supportive treatment with appropriate breathing exercises, as well as expectoration and strengthening nutrition can aid in weaning from ventilation. In this study, we aimed to describe a comprehensive approach including sequential invasive-noninvasive mechanical ventilation weaning and ERT for patients with weaning difficulty.

## Case presentation

### Subjects

The inclusion criteria for enrollment in the present study were: 1) Presence of the following clinical manifestations of the disease: cardiac hypertrophy or hypertrophic cardiomyopathy, progressive weakness of skeletal muscle, recurrent respiratory infection; 2) GAA activity in peripheral blood leukocytes lower than normal; 3) GSD II confirmed by GAA gene analysis; 4) Presence of difficulty in weaning, defined as [2, 33]: the need for more than three spontaneous breathing trials or more than 1 week for successful weaning achievement. Weaning success was defined as a patient’s ability to tolerate spontaneous breathing for more than 48 h; and 5) mechanical ventilator use duration longer than 14 days and difficulties in weaning from mechanical ventilation.

We retrospectively analyzed six difficult-to-wean GSD II patients at the pediatric intensive care unit (ICU) of the First Affiliated Hospital, Sun Yat-sen University from October 2015 to December 2017.

The patients’ detailed clinical data are presented below (Table 1).

**Table 1:** Clinical features and ERT of 6 children with GSD II

		P1	P2	P3	P4	P5	P6
Form		Juvenile-onset	Juvenile-onset	Juvenile-onset	Infantile-onset	Infantile-onset	Infantile-onset
Gender		F	M	F	M	M	M
Age of onset (mo)		15	20	47	5	1	4
Initial symptoms		Unsteady walking, recurrent respiratory infection	Walked slowly,recurrent respiratory infection	Generalized fatigue, slow running, hoarse voice, ptosis	Myocardial hypertrophy, delayed gross motor skills	Myocardial hypertrophy	Hypertrophic cardiomyopathy
Best gross motor milestone (age obtained in months)		Walk (15 → lost at 26)	Walk (20 → lost at 24)	Walk (14)	walk (13 → lost at 33)	stand with support (15)	MHC (4)
LVEdD (Z score>2) at baseline, age (mo)		✖	✓, 24	✖	✓, 5	✓, 1	✓,4
Age at first admission to our hospital (mo)		32	24	49	34	3	4
Weight at admission (kg)		10 (−2.2SD)	9 (−3.0SD)	12 (−2.2SD)	12 (−1.8SD)	5.2 (−2.0SD)	6.5 (−1.3SD)
Height at admission (cm)		87 (−1.6SD)	90 (−1SD)	95 (−2.3SD)	94 (−0.3SD)	58 (− 1.6SD)	63 (−0.6SD)
BMI at admission (kg/m^2^)		13.2	11.1	13.2	13.5	15.4	16.3
Dysphagia at baseline		✖	✖	✖	✖	✓	✓
Feeding pattern, nutrient type during hospitalization		NGT, peptamen	NGT,peptamen	Oral feeding, Normal diet	NGT,peptamen	NGT,formula milk	NGT,formula milk
Total calories (kcal/kg/day)		120–135	90–120	70–80	70–100	100–136	100–110
Rate of proteins with diet		12%	12%	10%	12%	15%	15%
Age of final diagnosis (mo)		31†	25†	49	7†	2†	5
Age of ERT intiation (mo)		32	31	56	34	3	5
Symptoms before starting ERT		Bed-ridden, muscle weakness of limbs, 24-h invasive ventilation	Bed-ridden, muscle weakness of limbs, 24-h invasive ventilation	Serious fatigue after exercises	Bed-ridden, myocardial hypertrophy, muscle weakness of limbs, 24-h invasive ventilation	Respiratory failure, 24-h invasive ventilation	Hypertrophic cardiomyopathy, respiratory failure, 24-h invasive ventilation
ERT Duration (mo, 20 mg/kg qow)		15.5	4.5	2	2.5	17	2
CRIM status		NT	NT	NT	NT	NT	NT
Age (mo); follow up status		72mo, wheelchair, tracheal intubation, 24-h mechanical ventilation	48mo, wheelchair, Weaned from the ventilator	96mo, went to school normally, physical exercise intolerable	36mo, died after abandoning treatment	21mo, walk independently, delayed gross motor skills	7mo, died after abandoning treatment
GAA activity in PBLC^b^		0.90	1.45	1.03	8.20	0.00	2.86
Genotype^c^		c.1082C>T,c.1935C>A (both potentially less severe)	c.1216G>A (unknow),c.1935C>A (potentially less severe)	c.1216G>A (unknow),c.1935C>A (potentially less severe)	c.1557G>A (unknown), c.1978C>T (potentially less severe)	homozygotic mutation of c.1935C>A (potentially less severe)	c.1822C>T (very severe), c.1935C>A (potentially less severe)
Age of initial MV (mo),Duration of IV (NIV) (mo)		30,36 (0.4)	23,1 (0)^a^	47,1 (0)^a^	31,5 (0)	3,3.5 (0.6)	5,2 (0.1)
Age of MV for the second time (mo),Duration of IV (NIV) (mo)		NA	30,2.5 (4)	72,1 (3)^a^	NA	15,1 (0.4)	NA
Trachcostomy (Yes/No)		Yes^d^	No	No	No	No	No
ABG pre-ventilation/worst	PH	7.16	7.13	7.33	7.22	7.27	7.33
	PO2 (mmHg)	84	88	65	74	39	808
	PCO2 (mmHg)	111	98	80	84	93	85
ABG on discharge/in the recovery phase	PH	7.39	7.48	7.41	7.42	7.35	7.44
	PO2 (mmHg)	80	62	93	97	72	89
	PCO2 (mmHg)	55	39	47	50	59	50

*GSD II* Glycogen storage disease type II, *P* patient no, *mo* months or month, *F* female, *M* male, *LVEdD* left-ventricular end-diastolic diameter, *MHC* minimal head control, *SD* standard deviation, *BMI* Body Mass Index, *NGT* naso-gastric tube, *ERT* enzyme replacement therapy, *CRIM* Cross reactive immunological material, *NT* not tested, *GAA* acid alpha- glucosidase, *PBLC* peripheral blood leukocytes, *MV* mechanical ventilation, *IV* invasive ventilation, *NIV* noninvasive ventilation, *NA* Not Applicable, *ABG* Arterial blood gas, *PH* Potential Hydrogen, *PaO2* Partial pressure of oxygen, *PaCO2* Partial pressure of carbon dioxide

^a^she or he was diagnosed in a previous hospital and successful withdraw of mechanical ventilation without enzyme replacement therapy in the first time

^b^normal GAA activity in PBLC: > 14 nmol/h/mg; ^c^ please visit www.pompecenter.nl for the severity of gene mutation

^d^she had a tracheotomy at 46 months old

**Patient 1**, a 32-month-old girl with the juvenile form of GSD II, was hospitalized with difficulties in weaning from the ventilator. She had suffered from muscular weakness since age 15 months, when she started walking unsteadily. She lost the ability to walk at 26 months. She was admitted to our hospital using a transport ventilator. She exhibited a repeated and weak cough, and was susceptible to recurrent chest infections. Oral sedative use was required for chest computed tomography performance. After sedation with 0.5 ml/kg (5 ml) of oral chloral hydrate, she developed respiratory muscle weakness and ventilator failure, and required invasive ventilation at age 30 months (Tables 1 and 2).

**Table 2:** Changes in respiratory and muscle strength of P1 (juvenile-onset) after enzyme replacement therapy

Months of ERT (28 days/month)	Proportion of ventilator time			MRC scale	
	Invasive BiPAP%	Invasive CPAP%	NIV% or time	UL	LL
0.0	100	0	10 min	3	3+
0.5	29	71	0	3	3+
1.0	21	79	30 min	4	3+
1.5	14	86	0	4+	3+
2.0	0	100	0	5-	4-
2.5–3.5	20	80	0	5-	4
4.0–5.0	0	100	0	5	5-
5.5^a^	79	21	2 h	5	5
6.0	7	93	0	5	5
6.5–7.0	0	100	0	5	5
7.5	14	86	2d	5	5
8.0–8.5	15	85	0	5	5
9.0	64	14	3d	5	5
9.5–10.5	6	94	0	5	5
11.0	57	0	6d	5	5
11.5–13.5	37	63	0	5	5
14.0–14.5^a^	100	0	0	5	5
15.0^b^	29	0	0	5	5

*ERT* enzyme replacement therapy, *P* patient no, *BiPAP* Biphasic positive airway pressure, *min* minutes, *hr* hours, *d* days, *CPAP* Continuous positive airway pressure, *NIV* noninvasive ventilator, *MRC* medical research council, *UL* Upper-limb muscle strength, *LL* lower-limb muscle strength

^a^Pulmonary infection; ^b^Tracheotomy

**Patient 2**, a 24-month-old boy with the juvenile form of GSD II, who presented with difficulties in weaning from the ventilator at age 23 months, was admitted to our hospital for the first time using a transport ventilator. He walked slowly and began having repeated respiratory infections at age 20 months. He was successfully withdrawn from mechanical ventilation without ERT at the first admission, because he was not diagnosed with GSD II. His symptoms improved, and he was discharged home. At age 30 months, he was admitted to our hospital again due to respiratory failure and required invasive ventilation again (Tables 1 and 3).

**Table 3:** Changes in respiratory and muscle strength of P 2 (juvenile-onset) after enzyme replacement therapy

Months of ERT (28 days/month)	Proportion of ventilator time			MRC scale	
	Invasive BIPAP%	Invasive CPAP%	NIV%	UL	LL
0.0	100	0	0	4	3
0.5	29	71	0	4	3+
1.0	21	79	0	4	3+
1.5	14	86	0	4+	3+
2.0	0	90	10	4+	3+
2.5	14	50	36	4+	3+
3.0	0	0	100	4+	3+
3.5	0	0	100	5-	3+
4.0	0	0	50^a^	5-	3+

*ERT* enzyme replacement therapy, *P* patient no, *BIPAP* Biphasic positive airway pressure, *CPAP* Continuous positive airway pressure, *NIV* noninvasive ventilation, *MRC* medical research council, *UL* Upper-limb muscle strength, *LL* lower-limb muscle strength

^a^at night

**Patient 3** with the juvenile form of GSD II is the older sister of Patient 2. She experienced generalized fatigue, showed bilateral ptosis, ran slowly, and spoke with a hoarse voice at age 47 months. She was transferred to our hospital at age 49 months due to pneumonia and respiratory failure from another hospital using a transport ventilator (Table 1).

**Patient 4**, a 34-month-old boy with the infantile form of GSD II, presented with pneumonia complicated with

respiratory failure and was admitted to our hospital using a transport ventilator. He had left ventricular hypertrophy at age 5 months, began having repeated respiratory infections at age 12 months, and while he could walk at 13 months, he lost the ability at 33 months (Table 1).

**Patient 5**, a 3-month-old boy with the infantile form of GSD II, presented with ventricular hypertrophy at age 1 month and had repeated respiratory infections when he was admitted to our hospital for the first time. His condition quickly evolved into pneumonia complicated with respiratory failure on day 3 after admission, requiring invasive ventilation. After 4 months of undergoing ERT, he was successfully weaned off mechanical ventilation. His symptoms improved, and he was discharged home at age 7 months. At age 15 months, he was admitted to our hospital again due to respiratory failure and required invasive ventilation again (Tables 1 and 4).

**Table 4:** Changes in respiratory and muscle strength of P5 (infantile-onset) after enzyme replacement therapy

Months of ERT (28 days/month)	Proportion of ventilator time		MRC scale	
	Invasive BIPAP%	NIV %	UL	LL
0.0	100	0	4-	4-
0.5–4.5	100	0	4-	4-
4.5–5.5	0	100	4	4
>4.5^a^	0	0	4+	4+

*ERT* enzyme replacement therapy, *P* patient no, *BIPAP* Biphasic positive airway pressure, *NIV* noninvasive ventilation, *MRC* medical research council, *UL* Upper-limb muscle strength, *LL* lower-limb muscle strength

^a^at home

**Patient 6**, a 4-month-old boy with the infantile form of GSD II, presented with hypertrophic cardiomyopathy and pneumonia complicated with respiratory failure and was admitted to our hospital. He required invasive ventilation on the day of admission (Table 1).

All six patients had a full-term normal delivery with no history of neonatal asphyxia. The parents of these patients did not have a consanguineous marriage and their mothers were all healthy with normal pregnancies and deliveries. Patients 2 and 3 belonged to the same family, and the other patients had no family history of neuromuscular disorders.

### Therapeutic methods

#### General treatment

The patients with pneumonia received empirical anti-infection therapy. The anti-infection therapeutic regimen was adjusted according to the disease etiology. The patients were kept in the semi Fowler’s position for ventilator-associated pneumonia prevention. Routine nursing measures and prone-position ventilation were performed. After watching live-action video and doing exercises following medical staff, the patients who were older than 2 years old, were encouraged to practice independent expectoration, and to perform breathing exercises and undergo respiratory muscle training with their parents. Enhanced enteral and part parenteral nutrition were administered. The nutritional goal of enteral nutrition without and with parenteral nutrition were reached at more than 70 kcal/kg/day. We used music therapy, increased the number of ICU visiting hours, enhanced patients’ comfort levels, and encouraged them to cooperate with the weaning efforts, so as to enhance their confidence in weaning.

#### Treatment of primary disease

All patients diagnosed with GSD II whose parents signed an informed consent form were included. Approval from the ethics committee of the First Affiliated Hospital, Sun Yat-sen University was obtained. ERT with recombinant human acid alpha-glucosidase (rhGAA, alglucosidase alfa, Myozyme®, Sanofi Genzyme) is available for the treatment of GSD II at our hospital. At 1–27 months post diagnosis, the patients received biweekly infusions of 20 mg/kg recombinant human α-1,4-glucosidase. Patients 1–6 received ERT for 15.5, 4.5, 2, 2.5, 17 and 2 months, respectively. Using the Medical Research Council (MRC) scale, muscle strength, as well as the ventilator mode and parameters were assessed at the baseline and 1 month after each ERT session. These patients were followed-up for a median of 7.25 months (range, 2 to 16 months).

#### Sequential invasive-noninvasive mechanical ventilation weaning strategy

All the patients underwent a routine procedure during ventilator weaning as follows: 1) Lung and systemic infection control without complications was achieved; 2) When the low parameter invasive biphasic positive airway pressure level was well-tolerated, the invasive continuous positive airway pressure (CPAP) mode was used. The indications for non-invasive mechanical ventilation were: 1) More than 12 h of invasive CPAP were tolerated [positive end-expiratory pressure (PEEP) ≤4 cm H_2_O; 2) pressure support ≤6 cm H_2_O]; 3) Arterial blood pH before weaning of 7.3–7.5 was achieved, as were a partial pressure of oxygen (PaO_2_) ≥60 mmHg and partial pressure of carbon dioxide (PaCO_2_) 30–55 mmHg; 4) When patients satisfied the above-stated conditions, noninvasive positive pressure ventilation was used immediately after extubation (sequential invasive-noninvasive strategy); 5) Weaning was performed in the morning, as long as the quality of sleep the previous night was good; 6) Patients were required to be conscious and able to independently cough and expectorate, and ERT was ideally used within the last week; and 7) A total of 1–2 mg/kg of methylprednisolone 20 min before weaning was intravenously provided to reduce the degree of laryngeal edema in patients who required invasive ventilation for longer than 1 month.

#### Matters needing attention during weaning from mechanical ventilation

1) Choosing an appropriately sized nasal mask or mouth-nose mask with good air tightness for patients; 2) Humidified ventilation use to improve sputum expectoration; 3) Administration of Budesonide and Salbutamol by drug aerosol inhalation for the alleviation of laryngeal edema and prevention of airway spasms; and 4) Close monitoring of vital signs and blood gas analysis after weaning.

### Criteria for weaning failure

1) Occurrence of respiratory distress and hemodynamic instability within a week after invasive ventilator use, with a respiratory rate greater than 70 breaths per minute or an increase of 30 breaths per minute, heart rate greater than 180 beats per minute or an increase of 20 beats per minute, and a change in systolic blood pressure ≥ 20%; 2) Arterial blood gas analysis showed a pH ≤7.30, arterial oxygen saturation (SaO_2_) ≤80%, PaCO_2_ > 75 mmHg, PaO_2_ < 60 mmHg or PaO_2_/Fraction of inspired oxygen (FiO_2_) < 200, or disturbance of consciousness.

## Results

With comprehensive treatment, the respiratory muscle strength of all patients was improved. Their nutritional status also improved after enhanced nutritional support was provided. Their detailed clinical data are shown in Tables 1, 2, 3, 4, 5.

**Table 5:** Cardiac structure and function after ERT in children with infantile GSD II

Months of ERT (28 days/month)	Age (months)			Interventricular septal thickness (mm)			Left ventricular ejection fraction(%)		
	P4^a^	P5	P6^a^	P4^a^	P5	P6^a^	P4^a^	P5	P6^a^
0.0	34	3	5	12	12	17	60	58	32
1.0	35	4	6	11	9	14	63	63	45
2.0	36	5	7	–	7	11	–	–	48
17.0	–	22	–	–	5	–	–	75	–

*ERT* enzyme replacement therapy, *GSD II* Glycogen storage disease type II, *P* patient no

^a^patient died after abandoning treatment; — untreated

**Patient 1:** All six attempts to wean from invasive ventilation and start non-invasive ventilation failed. The major manifestations of weaning failure included: (1) Body temperature ≥ 38.0 °C within 48 h after weaning from invasive ventilation; (2) Difficulty in expectoration with respiratory muscle fatigue, increased rale rates as observed on lung auscultation, and PaCO_2_ ≥ 60 mmHg; (3) Mental problems during ventilator-dependence, such as emotional stress, anxiety and depression; and (4) Tolerance achievement within 1–6 days of each non-invasive ventilation, upon which emergency intubation and mechanical ventilation were finally required. After six weaning failures, the patient underwent tracheal intubation and mechanical ventilation after more than 1 year (30 doses) of ERT administration. Before ERT, muscle strength testing using the MRC scale was performed, showing a score of 3 for the upper limb and 3+ for the lower limb. The patient could not sit. The muscle strength of her limbs improved after each ERT session, with the strength returning to normal after 5 months of ERT, and she could sit independently. Ventilator support was gradually reduced to half ventilator support through reductions in the invasive CPAP level after 2 months of ERT and then reductions in the PEEP level. Several minutes or hours of tracheal intubation in a spontaneous breathing state with oxygen supplementation were allocated every day to respiratory muscle training. From the time of disease onset, the patient received invasive mechanical ventilation for 30 months and intermittent non-invasive mechanical ventilation for 11 days (Tables 1 and 2).

**Patient 2:** The patient received invasive mechanical ventilation twice due to pneumonia. Using the sequential invasive-noninvasive strategy, he was successfully weaned from ventilation without ERT the first time (age 23 months) and after 3 months of ERT the second time (age 30 months). Before ERT, muscle strength testing using the MRC scale showed an upper limb score of 4 and lower limb score of 3. The patient could sit but was not very stable. He could not stand or walk. The muscle strength of his limbs improved after each ERT session; after 4 months of ERT, he showed an upper limb score of 5- and lower limb score of 3+. He could sit independently. From the time of disease onset, the patient received invasive mechanical ventilation for 3.5 months and intermittently required non-invasive mechanical ventilation at home at night (Tables 1 and 3).

**Patient 3**: She received invasive mechanical ventilation twice due to pneumonia. Using the sequential invasive-noninvasive strategy, mechanical ventilation was successfully withdrawn for the first time before diagnosis (age 49 months) and treatment with ERT (age 56 months). She received 2 months of ERT due to limb weakness when she was aged 56 months. She required invasive mechanical ventilation due to pneumonia and respiratory failure for the second time for a period of 27 days. She was also successfully weaned from ventilation without ERT this time (age 72 months). Her 6-min walk test result increased from 100 m at the baseline to 300 m after 2 months of ERT. From the time of disease onset, she received invasive mechanical ventilation for nearly 2 months and non-invasive mechanical ventilation for 3 months (Table 1).

**Patient 4:** He required invasive mechanical ventilation due to pneumonia and respiratory failure. Weaning failure from invasive ventilation was confirmed after 2.5 months of ERT, and he died after treatment abandonment (Tables 1 and 5).

**Patient 5:** ERT was initiated in this patient after diagnosis, and he required invasive mechanical ventilation twice due to pneumonia. Using the sequential invasive-noninvasive strategy, he was successfully weaned from ventilation the first (age 3 months) and second (age 15 months) times. From the time of disease onset, he received invasive mechanical ventilation for about 4.5 months and non-invasive mechanical ventilation for about 1 month. He demonstrated features of mild movement retardation. He was able to raise and support his head at age 9 months, sit independently at 12 months old, and stand with support at 15 months (Tables 1, 4 and 5).

**Patient 6:** He required invasive mechanical ventilation due to pneumonia and respiratory failure. He showed invasive ventilation weaning failure after 2 months of ERT and died after treatment abandonment (Tables 1 and 5).

## Discussion and conclusions

GSD II patients experience progressive muscle weakness, especially in the limbs and trunk, including in the muscles that control breathing, and may eventually require mechanical ventilation. Predictors of poor respiratory outcomes include male sex, disease duration, and overall neurological impairment [8, 9]. The typical manifestations of infantile-onset GSD II are cardiomegaly at an early stage [6], and quick progression to cardiopulmonary insufficiency. The incidence of pulmonary infection or dyspnea in infantile-onset GSD II is 78%; 29.2% (49/168) of such patients develop respiratory failure and require mechanical ventilation [10]. Broomfield et al. reported that 27% (9/33) of infantile-onset GSD II patients had respiratory failure and the need for long-term ventilation [11], which are associated with high morbidity and mortality. Juvenile-onset GSD II lacks major cardiac involvement and presents with slowly progressive myopathy of the limb-girdle muscles, trunk muscles, and diaphragm [12]. The typical manifestations of juvenile-onset GSD II are unsteady walking, recurrent pneumonia, and easily progression to respiratory failure requiring mechanical ventilation. Two juvenile-onset GSD II patients, as reported by Orlikowski et al. [13], who had severe respiratory symptoms at the baseline, remained on continuous ventilation support 24 h a day throughout the study. Once GSD II patients develop pneumonia and respiratory failure needing mechanical ventilation, it is very difficult to achieve weaning from mechanical ventilation. In GSD II, the disease severity depends on the disease type (infantile, juvenile or adult-onset) [12], the presence of cardiomyopathy, and the cross-reactive immunological material (CRIM) status [14].

ERT can improve muscle strength and respiratory function [15] and prevent the deterioration of respiratory function [16]. Infantile-onset GSD II usually presents with hypertrophic cardiomyopathy and such patients die before age 1 year, if not treated with ERT. For infantile-onset GSD II patients, the earlier ERT is administered the better [17]. Broomfield et al. [11] studied 33 United Kingdom patients with infantile-onset GSD II and found that 70% of them had radiological signs of focal pulmonary collapse and 27% (9/33) required mechanical ventilation. Chakrapani et al. [18] analyzed 20 infantile-onset GSD II patients who received ERT. These 20 patients were divided into two groups according to whether or not mechanical ventilation lasted longer than 2 weeks. All eight cases requiring ventilation for > 2 weeks eventually required long-term ventilation, and two of them died. Another study [19] showed that only 50% of ICU patients who were treated with mechanical ventilation for more than 14 days were successfully weaned off mechanical ventilation, and only 19% were discharged home. In our study, three patients with infantile-onset GSD II received mechanical ventilation for longer than 2 weeks. Patient 5 was successfully weaned from ventilation, and Patients 4 and 6 died.

Parini et al. [20] studied 28 infantile-onset GSD II patients from 13 Italian centres who started ERT within the first year of life and were followed for a median period of 71 months (5 years 11 months). This study confirmed the better outcome of the CRIM-positive patients but at the same time, showed the inability of the current therapeutic approach to reverse or stabilize the disease progression. So the long-term effectiveness of ERT is still not completely defined. Larger multicentre studies are needed as well as the development of new therapeutic approaches [20].

Deroma et al. [21] also studied eight Italian juvenile-onset GSD II patients treated with ERT for at least 72 months. The respiratory function of four patients showed a slight improvement and four patients remained stable; two of them already required ventilator support. In our study, three patients with juvenile-onset GSD II required mechanical ventilation. The respiratory function of three patients improved, one patient required tracheal intubation and mechanical ventilation at home, and two patients were successfully weaned from ventilation. Juvenile-onset GSD II patients have previously been reported on in individual case studies or small series, and our juvenile-onset GSD II patients cannot be compared to them, due to the vastly differing ages.

Falk et al. [22] showed that respiratory dysfunction in GSD II is mainly caused by significant pathological alterations at the neuromuscular junctions of the diaphragm as prominent features of disease pathology. Despite the cardiac and skeletal muscle, and force vital capacity improvements [18, 21, 23], slowly progressive neuromuscular weakness persists, which leads to diaphragmatic paralysis [24, 25]. In our study, most of the patients were successfully weaned from the ventilator after ERT. This shows that ERT can improve lung function. In Patient 1, in whom weaning from invasive mechanical ventilation failed all six times, diaphragm dysfunction may have been the cause. ICU-acquired respiratory muscle dysfunction [26] may have been another cause of unsuccessful weaning in Patient 1.

Smith et al. [27] found that a combined human trial of diaphragmatic gene therapy and inspiratory muscle conditioning exercise was beneficial to the dynamic motor function of the diaphragm in some children with infantile-onset GSD II. Todd et al. [28] found that correcting neuromuscular deficits with gene therapy may be essential to the successful correction of neuromuscular function in GSD II. Smith et al. [29] showed that neuromuscular activity via diaphragm pacing could promote weaning from mechanical ventilation in patients with Pompe disease who are unresponsive to conventional, muscle-directed treatment.

Remiche et al. [30] showed that there was no significant correlation between the level of GAA residual activity and the presence of a “severe” mutation on the second allele. Leukocyte or muscle GAA residual activity was not correlated to a protective effect (e.g., one patient had very low residual activity but her symptoms began presenting only at age 61 years) [30]. Previous research has shown [9] that male sex is a prognostic factor for poor respiratory outcomes. In our study, Patients 1 and 3 were female, with nearly the same GAA activity level. Patient 2, the younger brother of Patient 3, presented with severe respiratory involvement and weaning from mechanical ventilation proved more difficult than in his older sister. Patients 2 and 3 had the same genotype, but the younger brother who had higher GAA activity levels presented with severe respiratory involvement leading to more severe weaning difficulties. In addition, patient 5, who had zero GAA activity, was successfully weaned from mechanical ventilation twice. This shows that there maybe no significant correlation between severe clinical manifestations such as respiratory symptoms and GAA enzyme activity.

Enzyme therapy and immune response are related to a CRIM status [14, 20]. CRIM negative/positive with high levels of antibodies are a predictable sign of poor outcome in infantile form. CRIM-positive patients seem to have better clinical outcomes than CRIM-negative patients [14, 20], because their antibody titers are generally lower than those of CRIM-negative patients. Yang et al. [31] who reported on 14 infantile-onset patients who were CRIM-positive showed that 12/14 patients had the most commonly found mutation--c.1935 C > A. Juvenile form are by definition CRIM positive because they present residual enzymatic activity. However, none of our patients were tested for their CRIM status. We believe five of our six patients may have had a CRIM-positive status as they had the c.1935 C > A mutation [31]; this may be a reason for their better clinical outcomes.

Invasive mechanical ventilation is a lifesaving intervention for GSD II patients with respiratory failure. However, prolonged mechanical ventilation is associated with pulmonary complications, increased lengths of hospital stay, and higher mortality. Non-invasive ventilation is a weaning strategy for mechanical ventilation in patients with weaning difficulties. The Breathe Randomized Clinical Trial [32] showed that noninvasive ventilation could facilitate early liberation in patients in whom weaning from invasive mechanical ventilation was difficult. Non-invasive ventilation may reduce the durations of mechanical ventilation and ICU stays more effectively than conventional weaning. With the use of the sequential invasive-noninvasive mechanical ventilation weaning strategy, most of our patients were successfully weaned.

Patients requiring mechanical ventilation are at a risk for mental stress as they are aware that their ability to breathe totally depends on mechanical ventilation. Rose et al. [33] reported that 29% of prolonged mechanical ventilation patients were routinely referred for psychiatric/psychological treatment. Patients with emotional stress are likelier to experience weaning failure and death. Longer ICU visiting hours [34] and music therapy [35] can reduce the symptoms of anxiety and depression when weaning from mechanical ventilation. Efficient communication between patients and medical staff concerning the details of weaning from ventilation is essential in instilling confidence in the process. In our study, we provided all of the above to assist in weaning.

Recent studies [36–38] have shown that respiratory muscle training is an adjunctive treatment for respiratory weakness in late-onset GSD II. Malnutrition is associated with the duration of mechanical ventilation in critically ill children [39]. Malnutrition is a predictor of prolonged mechanical ventilation. All our patients had malnutrition, with body mass index values lower than 18 kg/m^2^; their nutritional status improved after enhanced nutritional support provision, which may have increased the tolerance to weaning from ventilation. We also used respiratory muscle training, which proved to be effective. Few articles have focused on weaning from mechanical ventilation in GSD II. ERT can improve muscle strength and respiratory function and prevent the deterioration of respiratory function, but it cannot reverse or stabilize disease progression in patients with weaning difficulties. We recommended a weaning strategy to guide clinicians to extubate patients who were difficult to wean to noninvasive ventilation, when the condition of weaning was met. The comprehensive treatment described in this paper, which included a sequential invasive-noninvasive mechanical ventilation weaning strategy and ERT in patients with weaning difficulties, was efficient and may be worth popularizing.

## Data Availability

All datasets generated for this study are included in the manuscript and the supplementary files.

## Abbreviations

ABG: Arterial blood gas
BiPAP: Biphasic positive airway pressure
BMI: Body Mass Index
CPAP: Continuous positive airway pressure
CRIM: Cross reactive immunological material
CT: Computed Tomography
ERT: Enzyme replacement therapy
F: Female
FiO_2_: Fraction of inspired oxygen
GAA: Acid alpha-glucosidase
GSD II: Glycogen storage disease type II
HFNC: High-flow nasal cannula
ICU: Intensive Care Unit
IV: Invasive ventilation
LL: Lower-limb muscle strength
LVEdD: Left-ventricular end-diastolic diameter
M: Male
MHC: Minimal head control
MRC: Medical Research Council
MV: Mechanical ventilation
NA: Not Applicable
NGT: Naso-gastric tube
NIV: Noninvasive ventilation
NT: Not tested
P: Patient no
PaCO_2_: Partial pressure of carbon dioxide
PaO_2_: Partial pressure of oxygen
PBLC: Peripheral blood leukocytes
PH: Potential Hydrogen
PS: Pressure support
SaO_2_: Arterial oxygen saturation
SD: Standard deviation
UL: Upper-limb muscle strength
